# Roburic Acid Targets TNF to Inhibit the NF-κB Signaling Pathway and Suppress Human Colorectal Cancer Cell Growth

**DOI:** 10.3389/fimmu.2022.853165

**Published:** 2022-02-09

**Authors:** Huanhuan Xu, Titi Liu, Jin Li, Fei Chen, Jing Xu, Lihong Hu, Li Jiang, Zemin Xiang, Xuanjun Wang, Jun Sheng

**Affiliations:** ^1^ Key Laboratory of Pu-er Tea Science, Ministry of Education, Yunnan Agricultural University, Kunming, China; ^2^ College of Science, Yunnan Agricultural University, Kunming, China; ^3^ State Key Laboratory for Conservation and Utilization of Bio-Resources in Yunnan, Yunnan Agricultural University, Kunming, China

**Keywords:** roburic acid, TNF, TNF-R1, NF-κB signaling, colorectal cancer

## Abstract

Tumor necrosis factor (TNF)-stimulated nuclear factor-kappa B (NF-κB) signaling plays very crucial roles in cancer development and progression, and represents a potential target for drug discovery. Roburic acid is a newly discovered tetracyclic triterpene acid isolated from oak galls and exhibits anti-inflammatory activity. However, whether roburic acid exerts antitumor effects through inhibition of TNF-induced NF-κB signaling remains unknown. Here, we demonstrated that roburic acid bound directly to TNF with high affinity (*K*_D_ = 7.066 μM), blocked the interaction between TNF and its receptor (TNF-R1), and significantly inhibited TNF-induced NF-κB activation. Roburic acid exhibited antitumor activity in numerous cancer cells and could effectively induce G0/G1 cell cycle arrest and apoptosis in colorectal cancer cells. Importantly, roburic acid inhibited the TNF-induced phosphorylation of IKKα/β, IκBα, and p65, degradation of IκBα, nuclear translocation of p65, and NF-κB-target gene expression, including that of XIAP, Mcl-1, and Survivin, in colorectal cancer cells. Moreover, roburic acid suppressed tumor growth by blocking NF-κB signaling in a xenograft nude mouse model of colorectal cancer. Taken together, our findings showed that roburic acid directly binds to TNF with high affinity, thereby disrupting its interaction with TNF-R1 and leading to the inhibition of the NF-κB signaling pathway, both *in vitro* and *in vivo*. The results indicated that roburic acid is a novel TNF-targeting therapeutics agent in colorectal cancer as well as other cancer types.

## Introduction

Colorectal cancer, a malignant disease of the digestive system, is the third most common cause of new cancer cases in both men and women and the second most frequent cause of cancer deaths (1, 2). Globally, approximately 1.4 million new cases of colorectal cancer are diagnosed and over 690,000 people die from this condition every year (3). The pathophysiology of colorectal cancer is very complex, and its development is a multistage process. Interactions between multiple genetic alterations, the host immune system, and environmental carcinogens have been implicated in the development of human colorectal cancer, which eventually leads to the uncontrolled growth of transformed cells and poor prognosis for patients (4, 5). Under normal conditions, surgical resection provides a possibility for cure in early-stage patients, whereas several anticancer drugs, such as oxaliplatin, 5-fluorouracil, and leucovorin, are recommended for treating advanced colorectal cancer (6, 7). However, current therapy regimens are not always effective at treating advanced colorectal cancer because of drug resistance and adverse side effects and toxicity (3). Consequently, there is an urgent need to identify potential therapeutic targets and discover drugs with greater specificity and less adverse effects from natural resources to treat colorectal cancer, as well as elucidate the underlying molecular mechanisms.

Considerable accumulated evidence has shown that chronic inflammation is closely associated with cancer development and progression (8). In particular, colorectal cancer patients exhibit extensive inflammatory infiltrates with high expression levels of cytokines in the tumor microenvironment (9). The proinflammatory cytokine tumor necrosis factor (TNF)-induced nuclear factor-kappa B (NF-κB) signaling pathway is the most intensively investigated pathway in most cell types (10). It represents a canonical NF-κB activation pathway that links inflammation and immunity to cancer development and progression and promotes tumorigenesis (8, 10, 11). Moreover, the TNF-induced NF-κB signaling pathway is a precisely regulated and therapeutically relevant pathway and a potential target for drug discovery (12, 13). The binding of TNF to its cognate receptor (TNF-R1) directly activates the canonical NF-κB signaling pathway, leading to the transcription of antiapoptotic genes (12, 14). Cancer cells can evade apoptosis *via* upregulating the expression levels of antiapoptotic proteins such as XIAP, Mcl-1, and Survivin (10). Consequently, there is increasing interest in identifying natural compounds that can inhibit this pathway (13, 15–17).

**Figure 1:**
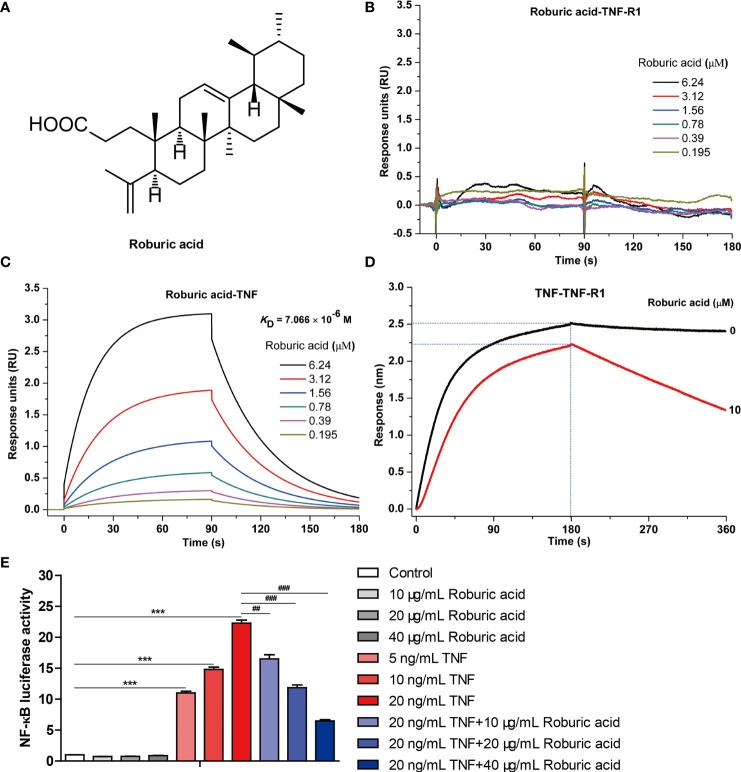
Roburic acid disrupts the interaction between TNF and TNF-R1 and blocks TNF-induced NF-κB activation. **(A)** The chemical structure of roburic acid. Direct interactions between roburic acid and TNF-R1 **(B)** and TNF **(C)** were detected using SPR analysis. **(D)** Roburic acid disrupts the interaction between TNF and TNF-R1, as determined by a BLI-based solution competition assay. **(E)** Roburic acid blocks TNF-induced NF-κB activation. Luciferase reporter assays were used to detect the NF-κB activity in 293-TNF Res (NF-κB) cells. Data are shown as means ± SEM of three independent replicates. ^***^ *P* < 0.001 compared with the control; ^##^ *P* < 0.01 and ^###^ *P* < 0.001 compared with TNF treatment only.

It is well known that new natural products isolated from medicinal plants are excellent and reliable sources of new anticancer drugs (16, 18–20). Roburic acid (molecular formula: C_30_H_48_O_2_; **Figure 1A**) is a newly identified tetracyclic triterpene acid originally isolated from oak galls, and later also found in *Gentiana macrophylla* Pall (21). Roburic acid has been shown to exert anti-inflammatory effects (21–23); however, whether it exhibits additionally bioactivities, especially anticancer effects, remains unknown. Given the anti-inflammatory properties of roburic acid and the critical role of TNF/TNF-R1-mediated NF-κB signaling in colorectal cancer development and progression, we speculated that roburic acid might disrupt TNF/TNF-R1-mediated NF-κB signaling and suppress the growth of human colorectal cancer cells. In the present study, we provide evidence that roburic acid binds to TNF with high affinity, which disrupts its interaction with TNF-R1 and leads to inhibition of the NF-κB signaling pathway, both *in vitro* and *in vivo*.

## Materials and Methods

### Chemicals and Reagents

Roburic acid of high purity grade (≥99%) was purchased from Chengdu Biopurify Phytochemicals Ltd (Chengdu, China). Purified cremophor (>98%) and crystal violet were purchased from Calbiochem, Inc. (San Diego, CA, USA) and Aladdin Bio-Chem Technology Co., Ltd (Shanghai, China), respectively. Dimethyl sulfoxide (DMSO) and 3-(4,5-dimethylthiazol-2-yl)-2,5-diphenyltetrazolium bromide (MTT) were obtained from Sigma-Aldrich (Saint Louis, MO, USA). RPMI-1640 medium (Gibco) and fetal bovine serum (FBS) were obtained from ThermoFisher Biochemical Products (Beijing) Co., Ltd and Biological Industries (Kibbutz Beit Haemek, Israel), respectively. Phosphate-buffered saline (PBS), a mixed penicillin–streptomycin solution (P/S), 4% paraformaldehyde, Triton X-100, antifade mounting medium with DAPI, radioactive immunoprecipitation assay (RIPA) buffer, phenylmethylsulfonyl fluoride (PMSF), and the nuclear protein extraction kit were purchased from Solarbio (Beijing, China). The BeyoClick EdU cell proliferation kit with Alexa Fluor 488 was purchased from Beyotime Biotechnology (Shanghai, China). Propidium Iodide (PI) and the Annexin V–FITC/PI detection kit were purchased from Beijing 4A Biotech Co., Ltd (Beijing, China). Anti-TNF-R1 antibody was purchased from HuaAn Biotechnology Co., Ltd (Hangzhou, China). Specific primary antibodies against Bcl-xL, Survivin, Cyclin B1, and Cyclin D1 were purchased from Abcam (Cambridge, MA, USA). Anti-Cyclin E1 and anti-XIAP antibodies were obtained from Santa Cruz Biotechnology (Santa Cruz, CA, USA). Antibodies against PARP, cleaved Caspase3, Caspase7, Caspase9, Bcl-2, Bax, Mcl-1, c-Myc, phospho (p)-IKKα/β, IKKα, IKKβ, p-IκBα, IκBα, p-p65 (Ser536), p65, p-JNK, JNK, p-ERK, ERK, p-p38, p38, p-AKT, AKT, p-STAT3, STAT3, Histone H3, β-tubulin, Ki-67, and rabbit IgG (H+L), F(ab’)_2_ Fragment (Alexa Fluor 594 conjugate) were purchased from Cell Signaling Technology (Beverly, MA, USA). Recombinant human TNF protein and horseradish peroxidase-conjugated secondary antibodies were obtained from R&D Systems (Minneapolis, MN, USA).

### Molecular Interaction Assay

Interactions between human TNF and TNF-R1 proteins and roburic acid were investigated using surface plasmon resonance (SPR) analysis. The human TNF soluble form (NP-000585.2) N-terminal fragment (Val 77–Leu 233) and the TNF-R1 (NP-001056.1) extracellular domain (Met 1–Thr 211) (Sino Biological Inc; Beijing, China) were prepared. SPR studies were performed using a Biacore S200 instrument (GE Healthcare, Sweden) at 25°C. Briefly, 50 μg/mL TNF-R1 and TNF in 10 mM sodium acetate buffer (pH 5.0) were respectively immobilized in flow cell-2 and -4 of the Series S CM5 Sensor Chip using an amine coupling kit (GE Healthcare), according to the standard Immobilization Wizard program. Roburic acid was double-diluted in PBS-P buffer (GE Healthcare) supplemented with 5% DMSO to concentrations ranging from 0.195 to 6.24 μM. The analytes were then injected to flow over the reference and active chip surfaces at a flow rate of 30 µL/min and the response units were measured. The association and dissociation times were both 90 s. The binding kinetics of TNF-R1 and TNF to roburic acid were analyzed with Biacore S200 Evaluation Software Version 1.1 using a 1:1 binding model.

In addition, solution competition biolayer interferometry (BLI) analysis was performed using an Octet Red 96 instrument (ForteBio, USA) at 25°C, as previously described (24). Briefly, TNF-R1 was biotinylated using amine-PEG3-biotin and then desalted using a Zeba Spin desalting column. The biotinylated TNF-R1 protein was loaded onto the surface of Super Streptavidin (SSA) biosensors (ForteBio). Roburic acid (0 or 20 μM) was preincubated with the immobilized TNF-R1 for 180 s. Subsequently, a 1 μM TNF solution supplemented with 0 or 20 μM roburic acid was allowed to interact with the immobilized TNF-R1 for 180 s, and dissociation was followed for 180 s in 0 or 20 μM roburic acid. Kinetic parameters and affinities were calculated from a non-linear global fit of the monitored binding curves using Octet Data Analysis software version 7.0 (Fortebio).

### Luciferase Reporter Assays

293-TNF Res (NF-κB) cell line purchased from Novoprotein Technology Co., Ltd (Shanghai, China) was used for luciferase reporter assays. The cells were cultured in serum free DMEM with or without roburic acid in the presence of TNF for 4 h. Luciferase activities were measured consecutively using the firefly luciferase reporter assay kit (Meilunbio, Dalian, China) with the GloMax^®^ 96 Microplate Luminometer (Promega, Madison, WI).

### Cell Lines and Cell Culture

All the cell lines (HCT-116, HCT-15, HT29, Colo205, SK-BR-3, BT549, BT-474, U251, 786-O, ACHN, A498, A549, NCI-H460, NCI-H226, NCI-H23, OVCAR-3, SK-OV-3, DU145, PC-3, and CCRF-CEM) used in this study were purchased from the American Type Culture Collection (ATCC; Manassas, VA, USA). All the cells were cultured in RPMI-1640 medium supplemented with 10% FBS and 1% P/S. Cells were cultured in an incubator (BINDER GmbH; Tuttlingen, Germany) with 95% air and 5% CO_2_ at 37°C.

### Cell Viability Assay

Cell viability was determined using the MTT method as previously described (25). For all the cancer cell lines, 2 × 10^4^ cells/well were seeded into 96-well plates and allowed to adhere for 24 h. The following day, the cells were treated with various concentrations of roburic acid (0–20 μM). After 48 h, MTT solution (5 mg/mL) was added to the cells. After 4 h, the supernatant was aspirated and 200 μL of DMSO was added to dissolve the formazan crystals. The optical density at 492 nm was then detected using a FlexStation 3 Multi-Mode Microplate Reader (Molecular Devices; Sunnyvale, CA, USA) and IC_50_ values were calculated.

### Colony Formation Assay

For clonogenicity analysis, 4 × 10^3^ viable HCT-116 and HCT-15 cells were seeded into 60-mm plates and incubated overnight. Then, vehicle or roburic acid was added to each plate at the respective concentrations (4, 8, or 16 μM) and the culture medium was changed every 2 days. After 8 days of incubation, the cells were fixed in 4% paraformaldehyde and then stained with a 1% crystal violet solution. The colonies were imaged and the number of colonies counted.

### Cell Proliferation Assay

HCT-116 and HCT-15 cells (5 × 10^4^ cells/well) were seeded into 12-well plates and allowed to adhere overnight. The following day, the cells were treated with various concentrations of roburic acid (0, 4, 8, or 16 μM) for 24 h. Then, HCT-116 and HCT-15 proliferation was determined using a BeyoClick EdU cell proliferation kit with Alexa Fluor 488 according to the manufacturer’s instructions. Briefly, the cells were treated with 10 μM EdU for 2 h, fixed in 4% paraformaldehyde for 20 min, washed twice with PBS, and permeabilized with 0.5% Triton X-100 for 30 min. Subsequently, the cells were incubated in 200 μL of click reaction buffer at room temperature for 30 min and then washed twice with PBS. Finally, the cells were mounted using antifade mounting medium with DAPI and observed under a fluorescence microscope (Leica). Images were captured at ×200 magnification and merged using ImageJ software (National Institutes of Health, Bethesda, MD, USA).

### Cell Cycle Analysis

HCT-116 and HCT-15 cells (2 × 10^5^ cells per plate) were seeded into 60-mm plates and incubated overnight. Subsequently, the cells were treated with various concentrations of roburic acid as described above. After incubation for 24 h, the cells were harvested, washed with PBS, and then fixed in 70% ethanol at −20°C overnight. After fixation, the cells were washed twice with pre-cooled PBS and incubated with binding buffer containing 100 μg/mL PI and 25 μg/mL RNase A in the dark for 30 min at 37°C. Finally, the fluorescence intensities of these samples were detected by BD FACSCalibur flow cytometry (BD Biosciences; San Jose, CA, USA) and FlowJo v.X.7.6.5 software was used to determine the cell cycle phase distributions.

### Cell Apoptosis Analysis

Cell apoptosis was analyzed using the Annexin V–FITC/PI detection kit according to the manufacturer’s instructions. Briefly, HCT-116 and HCT-15 cells (3 × 10^5^ cells per plate) were treated with different concentrations of roburic acid in serum-free medium for 24 h. The harvested cells were then incubated with 100 μL of binding buffer containing 5 μL of Annexin V–FITC and 10 μL of PI (20 μg/mL) in the dark for 5 min at room temperature. Subsequently, the prepared samples were analyzed by BD FACSCalibur flow cytometry within 1 h and the percentage of apoptotic cells was determined using FlowJo software.

### Immunoblotting Analysis

For protein extraction, after the corresponding treatments, HCT-116 and HCT-15 cells, as well as tumor tissues, were washed twice with pre-cooled PBS and lysed on ice using RIPA lysis buffer containing 1 mM PMSF. Cell lysates were quantified using an Enhanced BCA Protein Assay Kit (Beyotime Biotechnology) according to the manufacturer’s protocol. Subsequently, equal amounts of proteins were separated by 10% SDS–PAGE and electrophoretically transferred onto polyvinylidene fluoride (PVDF) membranes (Millipore). After blocking with 5% nonfat milk in PBST, the membranes were incubated with diluted specific primary antibodies at 4°C overnight, followed by incubation with the corresponding rabbit or mouse IgG horseradish peroxidase-conjugated secondary antibodies (diluted 1:5,000). Protein bands were detected with an Ultra-sensitive Enhanced Chemiluminescent Substrate Kit (4A Biotech) and visualized using a FluorChem E System (ProteinSimple, San Jose, CA, USA). The protein expression level was detected using AlphaView software (Cell Biosciences, Santa Clara, CA, USA).

### Nuclear and Cytoplasmic Protein Extraction

HCT-116 and HCT-15 cells (2.5 × 10^6^ cells per plate) were seeded into 100-mm plates and allowed to adhere overnight. The following day, cells were cultured in serum-free medium for 12 h and then pretreated with or without roburic acid (8 μM) for 4 h, followed by stimulation with TNF (10 ng/mL) for the indicated times. A nuclear protein extraction kit was used to isolate and extract nuclear and cytoplasmic proteins, according to the manufacturer’s protocol. In brief, the collected cells were incubated in cytoplasm lysis buffer containing 1 mM PMSF on ice for 10 min. The supernatants collected after centrifugation at 16,000 × *g* for 10 min at 4°C were considered cytoplasmic protein extracts. The pellets were then dissolved in nuclear lysis buffer containing 1 mM PMSF on ice for 10 min, and the supernatants obtained by centrifuging were regarded as the nuclear protein fractions. Finally, the cytoplasmic and nuclear protein extracts were subjected to immunoblotting analysis with the corresponding primary antibodies.

### Immunofluorescence Staining

HCT-116 and HCT-15 cells (5 × 10^4^ cells/well) were seeded into 12-well plates and allowed to adhere overnight. Cells were treated with or without roburic acid (8 μM) for 4 h and subsequently stimulated with TNF (10 ng/mL) for 30 min in serum-free medium. Then, the cells were washed twice with pre-cooled PBS, fixed in 4% paraformaldehyde for 20 min, washed twice with PBS, blocked with 1% bovine serum albumin (BSA) for 1 h at room temperature, incubated with anti-p65 antibody at 4°C overnight, gently washed twice with PBS, and incubated with Alexa Fluor 594-conjugated secondary antibody for 1 h at temperature in the dark. Subsequently, the slides were washed twice with PBS and mounted using antifade mounting medium with DAPI. Images were taken at ×400 magnification using a fluorescence microscope, and merged using ImageJ software.

### *In Vivo* Xenograft Studies

All animal experiments were carried out in accordance with the National Institutes of Health’s Guidelines for the Care and Use of Laboratory Animals and were approved by the Yunnan Agricultural University institutional ethics committee. Care was taken to minimize the discomfort, distress, and pain of the experimental animals. Eighteen 5-week-old male BALB/c nude mice were purchased from Cawens Lab Animal Co. (Changzhou, China) and allowed to acclimate for 1 week. The animals were housed in polypropylene cages with sterile paddy husk and were maintained under standard pathogen-free conditions (ambient temperature 24 ± 1°C, humidity 50–60%, 12 h light/dark cycle) with free access to a standard laboratory diet and water. HCT-116 (5 × 10^6^) and HCT-15 (4 × 10^6^) cells were harvested and suspended in 200 μL of a physiological saline solution, and injected subcutaneously into the left and right flanks of each mouse, respectively. One week after xenotransplantation, mice with tumors of approximately 100 mm^3^ were randomly and averagely divided into three groups. The tumor-bearing mice started daily intraperitoneal injection with either a vehicle (10% DMSO, 70% cremophor/ethanol (3:1), and 20% PBS) as previously described (18), or 5 or 10 mg/kg body weight roburic acid for 18 days. Body weight was recorded and tumor size was determined using electronic calipers every 2 days, and tumor volume was calculated in an unblinded manner according to the formula (*L* × *W* ^2^) × 0.5, where *L* is the length and *W* the width. At the end of the treatments, mice were euthanized by cervical dislocation and the isolated tumors were weighed, photographed, and used for immunoblotting analysis and immunohistochemical staining.

### Immunohistochemical Staining

Immunohistochemistry was performed according to standard methods. Paraffin-embedded xenograft tumor tissues were cut into 3-μm sections, deparaffinized, and rehydrated in different percentages (100, 95, 85, 75, and 65%) of ethanol. For antigen retrieval and to quench endogenous peroxidases, sections were incubated with 10 mM citric buffer (pH 6.0) and BLOXALL Blocking Solution (Vector Laboratories; Burlingame, CA, USA), respectively. The slides were incubated overnight at 4°C with primary antibodies against p-p65, cleaved Caspase3, and Ki-67. Detection was performed using the VECTASTAIN Elite ABC-Peroxidase Kit (Vector Laboratories) and an Enhanced HRP–DAB Chromogenic Kit (TIANGEN Biotech Co., Ltd; Beijing, China) according to the manufacturer’s instructions, followed by counterstaining with Mayer’s hematoxylin (Sigma–Aldrich). The slides were dehydrated with different concentrations of ethanol (65, 75, 85, 95, and 100%) and then mounted in Permount Mounting Medium (Fisher Scientific). Images were captured at ×400 magnification under a CKX41 microscope (Olympus, Tokyo, Japan).

### Statistical Analysis

Data are shown as means ± the standard error of the mean (SEM). All experiments were performed at least three times and representative images are shown. The Student’s *t*-test was performed using SPSS v17.0 (IBM, Armonk, NY, USA). A value of *P* < 0.05 was considered significant. The statistical parameters of quantitative data are reported in the corresponding figure legends.

## Results

### Roburic Acid Disrupts the Interaction Between TNF and TNF-R1 and Blocks TNF-Induced NF-κB Activation

Protein-protein interactions between TNF and its receptor TNF-R1 are known to regulate the canonical TNF-induced NF-κB pathway and are therefore considered a pivotal therapeutic target for the treatment of TNF-associated autoimmune diseases, including cancer (10, 26, 27). Given that roburic acid can exhibit anti-inflammatory activity and the underlying molecular mechanism is still unknown (21, 23), we investigated whether roburic acid could directly disrupt the interaction between TNF and TNF-R1.

We first detected the direct interactions between roburic acid and TNF-R1 and TNF using SPR analysis. The results showed that roburic acid could directly interact with TNF, but not with TNF-R1 (**Figures 1B, C** **)**. The apparent association (*K*_on_) and dissociation (*K*_off_) constants of roburic acid were calculated as 4.21 × 10^3^ M^−1^s^−1^ and 2.97 × 10^−2^ s^−1^, respectively, and the TNF-binding affinity (*K*_D_) was calculated as 7.066 μM, suggesting that the TNF-roburic acid complex is relatively stable.

Importantly, the BLI-based solution competition assay showed that roburic acid could compete with TNF-R1 for TNF binding (**Figure 1D**), indicating that roburic acid disrupts the interaction between the two proteins. Analysis of the intrinsic kinetic parameters indicated that roburic acid inhibited the association between TNF and TNF-R1, with the *K*_on_ value decreasing from 2.94 × 10^4^ to 1.30 × 10^4^ M^−1^s^−1^; roburic acid also promoted the dissociation between TNF and TNF-R1, with the *K*_off_ value increasing from 2.16 × 10^−4^ to 2.74 × 10^−3^ s^−1^. This demonstrated that roburic acid disrupted the interaction between TNF and TNF-R1, with the binding affinity (*K*_D_) decreasing from 7.33 × 10^−9^ to 2.11 × 10^−7^ M.

To further identify whether roburic acid could inhibit TNF-induced NF-κB activation, we directly performed NF-κB luciferase reporter assays *in vitro*. TNF significantly increased the NF-κB luciferase activity in a concentration-dependent manner, and roburic acid significantly blocked TNF-induced NF-κB activation in a concentration-dependent manner (**Figure 1E**).

Collectively, these results indicated that roburic acid directly binds to TNF with high affinity, blocks the interaction between TNF and TNF-R1, and inhibits TNF-induced NF-κB activation. This demonstrates that TNF is a direct target of roburic acid in the inhibition of the TNF-induced NF-κB activation, which prompted us to investigate whether roburic acid exerts antitumor effects through inhibition of TNF-induced NF-κB signaling.

### The HCT-116 and HCT-15 Cell Lines Showed the Greatest Sensitivity to Roburic Acid Treatment

**Figure 2:**
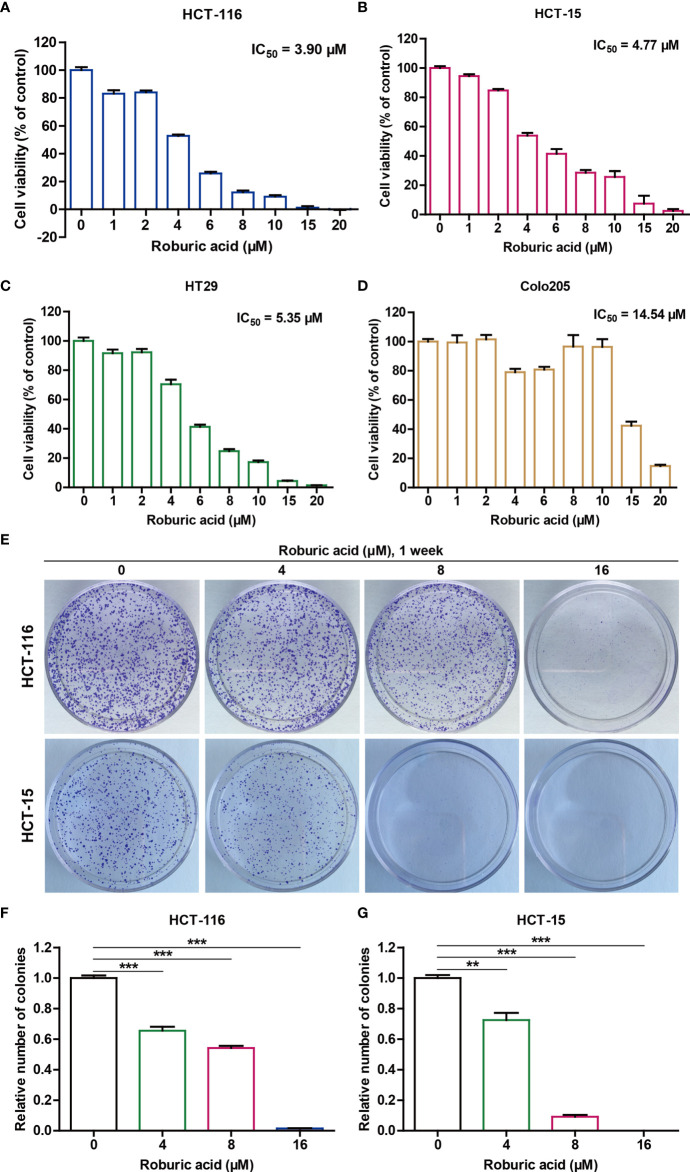
Roburic acid inhibits the viability of human colorectal cancer cells. **(A**–**D)** The cytotoxicity of roburic acid against human colorectal cancer cells. HCT-116 **(A)**, HCT-15 **(B)**, HT29 **(C)**, and Colo205 **(D)** cells were treated with roburic acid at the indicated concentrations for 48 h. An MTT assay was performed to evaluate cell viability and the IC_50_ values were calculated. **(E)** Flat plate colony formation assay of HCT-116 and HCT-15 cells treated with various concentrations of roburic acid (4, 8, or 16 μM). **(F, G)** Colony formation rates of the HCT-116 **(F)** and HCT-15 **(G)** cells are expressed as fold change. Representative images are displayed. Data are shown as means ± SEM of three independent replicates. ^**^ *P* < 0.01 and ^***^ *P* < 0.001 compared with the control.

To evaluate the potential cytotoxicity of roburic acid against human colorectal cancer cells, HCT-116, HCT-15, HT29, and Colo205 cells were treated with various concentrations of roburic acid for 2 days *in vitro* and cell viability was determined by the standard MTT method. As shown in **Figures 2A–D**, roburic acid greatly inhibited the viability of HCT-116, HCT-15, HT29, and Colo205 cells, with half-maximal inhibitory concentration (IC_50_) values of 3.90, 4.77, 5.35, and 14.54 μM, respectively. To further investigate whether roburic acid exhibits cytotoxicity against other types of cancer cells, we determined the IC_50_ values for three breast cancer cell lines (SK-BR-3, BT549, and BT-474), one central nervous system cancer cell line (U251), three kidney cancer cell lines (786-O, ACHN, and A498), four lung cancer cell lines (A549, NCI-H460, NCI-H226, and NCI-H23), two ovarian cancer cell lines (OVCAR-3 and SK-OV-3), two prostate cancer cell lines (DU145 and PC-3), and one leukemia cancer cell line (CCRF-CEM). As shown in **Supplementary Table S1**, the IC_50_ values for roburic acid ranged from 5–15 μM in these seven human cancer cell types. Taken together, the above results indicated that the HCT-116 and HCT-15 cell lines were the most sensitive to roburic acid cytotoxicity.

Notably, colorectal cancer has been shown to exhibit extensive inflammatory infiltrates with high levels of cytokine expression in the tumor microenvironment and TNF can activate NF-κB to promote colorectal cancer cell growth (9). Consistently, the high expression levels of TNF-R1 were detected in the HCT-116 and HCT-15 cell lines and TNF could effectively promote the growth of these cells in a concentration-dependent manner (**Supplementary Figure S1**). Therefore, we selected HCT-116 and HCT-15 cell lines for further studies. As expected, the flat plate colony formation assays showed that roburic acid could significantly inhibit HCT-116 and HCT-15 colony formation in a concentration-dependent manner (**Figures 2E–G**).

### Roburic Acid Suppressed DNA Synthesis in HCT-116 and HCT-15 Cells

To provide direct evidence for the cytotoxic response to roburic acid treatment in human colorectal cancer cell lines, we further investigated whether roburic acid could inhibit DNA synthesis in these cells. EdU incorporation assays showed that roburic acid treatment (4–16 μM) markedly suppressed DNA synthesis in both colorectal cancer cell lines (**Supplementary Figure S2**).

### Roburic Acid Treatment Triggered G0/G1 Cell Cycle Arrest in HCT-116 and HCT-15 Cells

**Figure 3:**
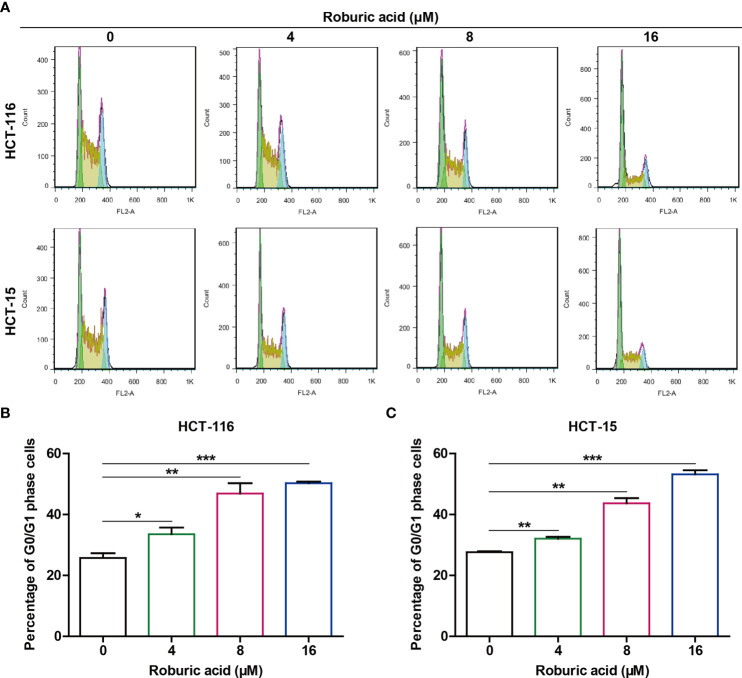
Roburic acid induces G0/G1 cell cycle arrest in HCT-116 and HCT-15 cells. **(A)** HCT-116 and HCT-15 cells were treated with roburic acid (4, 8, or 16 μM) for 24 h, the cell cycle phase distribution was assessed by BD FACSCalibur flow cytometry. **(B, C)** The percentage of G0/G1-phase cells in HCT-116 **(B)** and HCT-15 **(C)** cells was evaluated using FlowJo v.X.7.6.5 software. Representative images are displayed. Data are shown as means ± SEM of three independent replicates. ^*^ *P* < 0.05, ^**^ *P* < 0.01 and ^***^ *P* < 0.001 compared with the control.

Given that roburic acid could effectively suppress DNA synthesis in colorectal cancer cells, we speculated that it might induce cell cycle arrest. To test this, we treated HCT-116 and HCT-15 cells with different concentrations of roburic acid (4–16 μM) for 24 h and detected the cell cycle phase distribution using flow cytometry. As expected, roburic acid significantly increased the percentage of G0/G1 phase cells in both cell lines, and this effect was concentration-dependent (**Figure 3**). In addition, the percentages of cells in the S and G2 phases were significantly decreased in roburic acid-treated colorectal cancer cells (data not shown).

### Roburic Acid Triggered Apoptosis in HCT-116 and HCT-15 Cells

**Figure 4:**
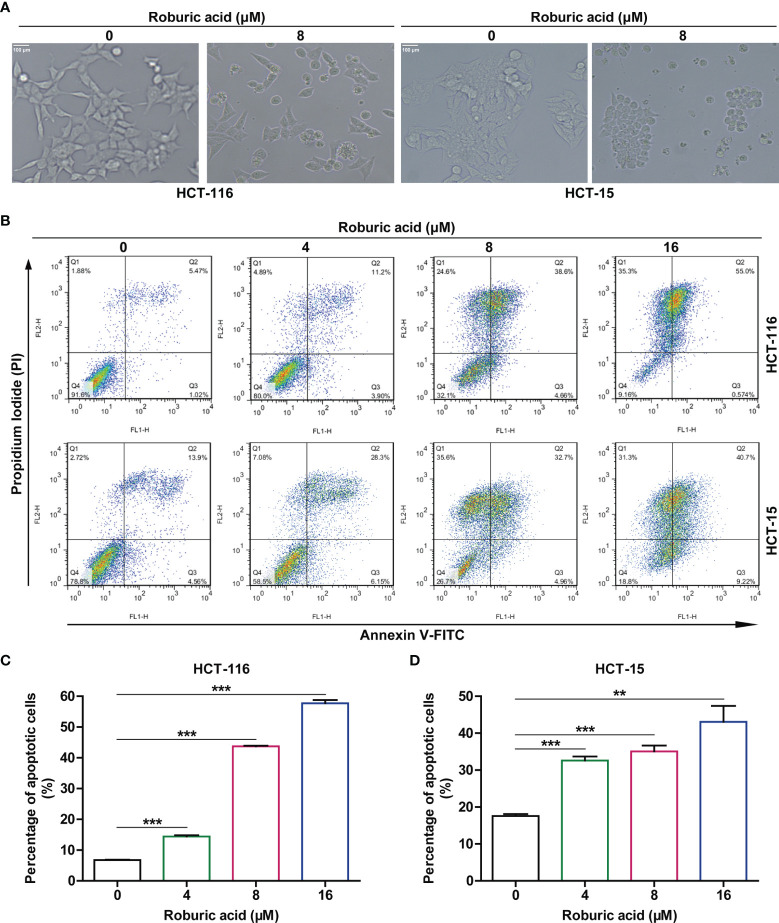
Roburic acid induces apoptosis in HCT-116 and HCT-15 cells. **(A)** The cell morphology of HCT-116 and HCT-15 cells changed markedly after treatment with roburic acid (8 μM) for 24 h (original magnification ×200). **(B)** HCT-116 and HCT-15 cells were treated with roburic acid (4, 8, or 16 μM) in serum-free medium for 24 h, and then stained with Annexin V–FITC/PI and analyzed by flow cytometry. **(C, D)** The percentages of Annexin V–FITC-positive apoptotic HCT-116 **(C)** and HCT-15 **(D)** cells were evaluated using FlowJo software. Representative images are displayed. Data are shown as means ± SEM of three independent replicates. ^**^ *P* < 0.01 and ^***^ *P* < 0.001 compared with the control.

Because we found that roburic acid could significantly inhibit the viability of HCT-116 and HCT-15 cells within 2 days, we speculated that roburic acid triggers cell death in addition to disrupting cell cycle distribution. Interestingly, we observed that HCT-116 and HCT-15 cells treated with roburic acid (8 μM) for 24 h exhibited a shrunk morphology, detached, and died (**Figure 4A**). We further determined the proapoptotic effect of roburic acid on HCT-116 and HCT-15 cells using Annexin V–FITC/PI staining and flow cytometric analysis. After treatment with roburic acid (4–16 μM) for 24 h, the number of Annexin V–FITC-positive (apoptotic) HCT-116 and HCT-15 cells exhibited a significant, concentration-dependent increase (**Figures 4B–D**). These results demonstrated that roburic acid triggers cell death through the induction of cell cycle arrest and apoptosis.

### Roburic Acid Modulated the Expression Levels of Multiple Cell Cycle- and Apoptosis-Related Regulators in Colorectal Cancer Cells

Because roburic acid markedly induced cell cycle arrest and apoptosis in colorectal cancer cells, we further investigated the protein levels of multiple cell cycle- and apoptosis-related regulators in HCT-116 and HCT-15 cells treated with roburic acid (4–16 μM) for 24 h. As expected, roburic acid treatment led to a significant decrease in the protein expression levels of cell cycle-related markers, including Cyclin B1, Cyclin D1, and Cyclin E1, in both HCT-116 and HCT-15 cells (**Supplementary Figure S3**). In addition, roburic acid treatment greatly enhanced the cleavage of poly (ADP-ribose) polymerase (PARP), Caspase3, Caspase7, and Caspase9 in these colorectal cancer cells in a concentration-dependent manner (**Supplementary Figure S3**). Importantly, roburic acid treatment also reduced the expression levels of several antiapoptotic proteins in both cell lines, including that of Bcl-2, Bcl-xL, XIAP, Mcl-1, and Survivin, in a concentration-dependent manner (**Supplementary Figure S3**). c-Myc is an oncogenic transcription factor that is highly expressed through different mechanisms in many cancer types, and is closely associated with promotion of the transition from the G0/G1 phase to the S phase of the cell cycle (28). Interestingly, we found that treatment with roburic acid significantly decreased the protein expression level of c-Myc in human colorectal cancer cells in a concentration-dependent manner (**Supplementary Figure S3**).

### Roburic Acid Inhibited the TNF-Induced NF-κB Signaling Pathway in Colorectal Cancer Cells

Several studies have indicated that roburic acid inhibits the NF-κB and MAPK signaling pathways and exerts anti-inflammatory effects (21, 23). Recently, it has become clear that TNF-induced NF-κB signaling also plays a critical role in colorectal cancer development and progression, and is a potential therapeutic target for the treatment of these conditions (9, 29, 30). Notably, pathways that activate NF-κB signaling can inhibit apoptosis through upregulation of the expression of antiapoptotic proteins such as XIAP, Mcl-1, and Survivin (16).

**Figure 5:**
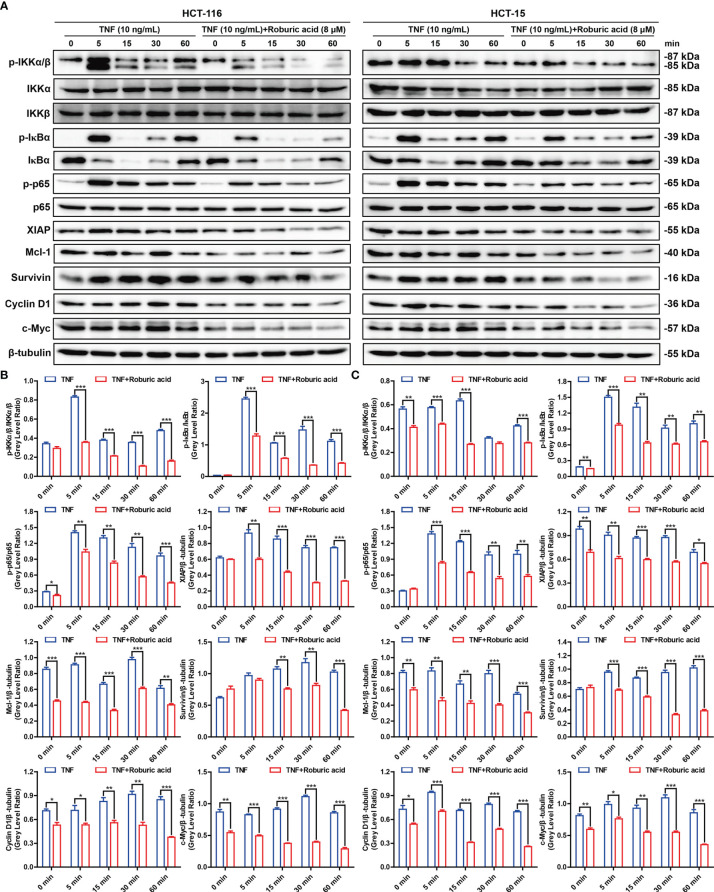
Roburic acid inhibits the TNF-induced NF-κB signaling pathway and antiapoptotic protein expression in colorectal cancer cells. **(A)** HCT-116 and HCT-15 cells were cultured in serum-free medium for 12 h and then pretreated with or without roburic acid (8 μM) for 4 h, followed by stimulation with TNF (10 ng/mL) for the indicated times. The cell lysates were used for immunoblotting analysis to measure the expression levels of the indicated proteins. Beta-tubulin was used as the loading control. The gray densities of the bands corresponding to the indicated proteins in HCT-116 **(B)** and HCT-15 **(C)** cells were quantified using AlphaView software. Representative images are displayed. Data are shown as means ± SEM of three independent replicates. Asterisks indicate significant differences compared with the TNF treatment at the same time point (^*^ *P* < 0.05, ^**^ *P* < 0.01, and ^***^ *P* < 0.001).

To further explore the molecular mechanisms by which roburic acid suppresses the expression of antiapoptotic proteins, we investigated whether roburic acid could block TNF-induced NF-κB signaling. As expected, TNF (10 ng/mL) treatment led to a marked activation of the NF-κB signaling pathway in both HCT-116 and HCT-15 cells, as determined by the observed increase in the levels of IKKα/β, IκBα, and p65 phosphorylation, degradation of IκBα, and induction of the protein expression of XIAP, Mcl-1, and Survivin (**Figure 5**). However, these effects were significantly inhibited by roburic acid treatment (8 μM) (**Figure 5**), which was consistent with the results of the BLI-based solution competition assay and NF-κB luciferase reporter assays (**Figures 1D, E** **)**. Notably, roburic acid treatment had no effect on the protein expression level of p65 in either cell line (**Figure 5**).

NF-κB can transactivate the expression of Cyclin D1 and c-Myc, which promotes cell proliferation, and suppress the expression of the proliferation factor JNK (10). As expected, roburic acid treatment significantly suppressed the expression of Cyclin D1 and c-Myc (**Figure 5**), and increased the levels of phosphorylated JNK in both HCT-116 and HCT-15 cells (**Supplementary Figure S4**). Moreover, the TNF-stimulated phosphorylation of ERK, p38, AKT, and STAT3 was decreased in both colorectal cancer cell lines (**Supplementary Figure S4**). Collectively, these results indicated that roburic acid inhibits TNF-induced NF-κB signaling in colorectal cancer cells.

### Roburic Acid Inhibited TNF-Induced P65 Nuclear Translocation in Colorectal Cancer Cells

**Figure 6:**
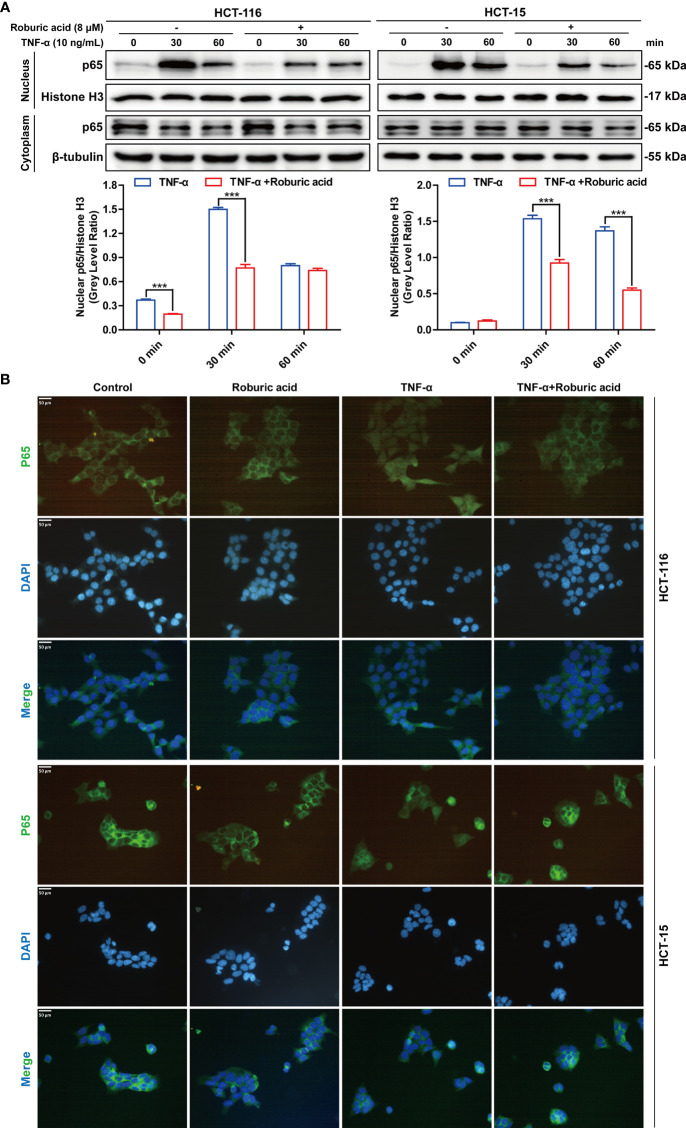
Roburic acid inhibits TNF-induced p65 nuclear translocation in colorectal cancer cells. **(A)** HCT-116 and HCT-15 cells were treated with roburic acid and TNF as described in **Figure 5**, except that the TNF treatment time points were different. Nuclear and cytoplasmic fractions were isolated and subjected to western blotting using an anti-p65 antibody. Beta-tubulin and Histone H3 were respectively used as loading control for the nuclear and cytoplasmic fractions. The gray densities of the bands corresponding to the indicated proteins in HCT-116 and HCT-15 cells were quantified using AlphaView software. **(B)** p65 nuclear translocation in HCT-116 and HCT-15 cells was determined by immunofluorescence staining. HCT-116 and HCT-15 cells were treated with TNF for 30 min. p65 was detected using the corresponding primary antibody and nuclei were stained with DAPI. The corresponding images (original magnification ×400) were merged using ImageJ software. Representative images are displayed. Data are shown as means ± SEM of three independent replicates. Asterisks indicate significant differences compared with the TNF treatment at the same time point (^***^ *P* < 0.001).

Nuclear translocation of p65 is a pivotal event in TNF-induced NF-κB pathway activation. We further investigated whether roburic acid could block the TNF-stimulated p65 nuclear translocation in HCT-116 and HCT-15 cells. As expected, roburic acid significantly inhibited p65 nuclear translocation at 0 to 30 min in HCT-116 cells and at 30 to 60 min in HCT-15 cells (**Figure 6A**). To further confirm this result, we performed immunofluorescence staining for p65 in HCT-116 and HCT-15 cells. As shown in **Figure 6B**, TNF (10 ng/mL) treatment greatly stimulated p65 nuclear translocation in both cell lines at 30 min; however, this effect was significantly inhibited by roburic acid (8 μM).

### Roburic Acid Reduced Tumor Growth by Blocking NF-κB Signaling in a Xenograft Mouse Model of Colorectal Cancer

**Figure 7:**
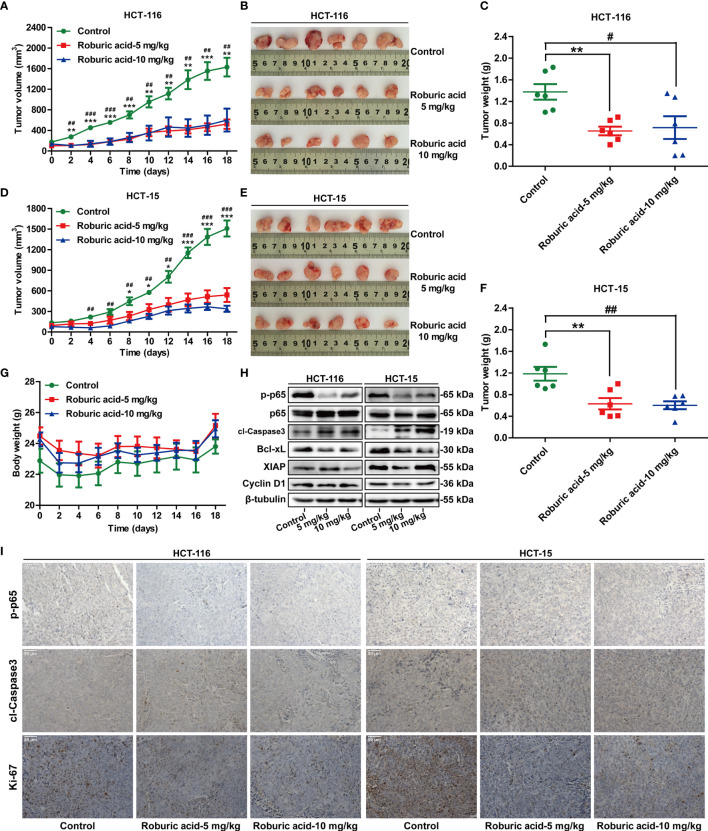
Roburic acid inhibits tumor growth by blocking NF-κB signaling in a xenograft mouse model of colorectal cancer. Roburic acid treatment inhibits tumor growth and weight of HCT-116 **(A**–**C)** and HCT-15 **(D**–**F)** human colorectal cancer xenografts in nude mice, but does not affect the body weight **(G)** of either group. **(H)** Tumor tissues from HCT-116 and HCT-15 xenografts were used for immunoblotting analysis to assess the protein expression of p-p65, p65, cleaved Caspase3 (cl-Caspase3), Bcl-xL, XIAP, and Cyclin D1. Beta-tubulin was used as loading control. **(I)** Paraffin-embedded HCT-116 and HCT-15 tumor tissue sections were immunostained with antibodies against p-p65, cl-Caspase3, and Ki-67 (original magnification ×400). Representative images are displayed. Data are shown as means ± SEM of six mice per group. ^*^ *P* < 0.05, ^**^ *P* < 0.01, and ^***^ *P* < 0.001, low-dose roburic acid group *vs* the control group; ^#^ *P* < 0.05, ^##^ *P* < 0.01, and ^###^ *P* < 0.001, high-dose roburic acid group *vs* the control group.

Having established the inhibitory effects of roburic acid on human colorectal cancer cells *in vitro*, we next investigated whether roburic acid could suppress cancer cell growth *in vivo* using a xenograft mouse model of colorectal cancer. Initially, HCT-116 and HCT-15 cells were injected subcutaneously into nude mice. One week after the xenotransplantation, the tumor-bearing mice were randomized into three groups and treated as described in the “Materials and methods” section. We found that roburic acid treatment effectively suppressed the growth of the xenografted colorectal tumors, as indicated by the significantly decreased tumor volume and weight (**Figures 7A–F**), and was consistent with the results of the *in vitro* experiments. In addition, treatment with roburic acid at the concentrations tested over 18 days did not affect the body weight of mice (**Figure 7G**), suggesting that roburic acid treatment had no side effects at the tested concentrations.

To further investigate whether roburic acid suppresses tumor growth by inhibiting the NF-κB signaling pathway *in vivo*, we performed western blotting analysis and immunohistochemical staining for the xenografted HCT-116 and HCT-15 tumor tissues. Consistent with the molecular findings *in vitro*, western blotting analysis showed that treatment with roburic acid inhibited the phosphorylation of p65, promoted the cleavage of Caspase3, and suppressed the protein expression of Bcl-xL, XIAP, and Cyclin D1 in the xenografted colorectal tumor tissues (**Figure 7H**). Furthermore, immunohistochemical staining of the tumor sections indicated that roburic acid treatment downregulated the expression of p-p65, while increasing the level of cleaved Caspase3 (**Figure 7I** and **Supplementary Figure S5**). These results were consistent with those of the immunoblotting analysis. Ki-67 is a specific marker for cell proliferation *in vivo*. As expected, we found that the expression of Ki-67 was decreased in the roburic acid-treated group compared with the control group (**Figure 7I** and **Supplementary Figure S5**). Taken together, these results demonstrated that roburic acid can suppress tumor growth by blocking NF-κB signaling in a xenograft mouse model of colorectal cancer.

## Discussion

Cancer remains a major public health problem worldwide, affecting millions of individuals and resulting in extensive morbidity and mortality (31–33). Approximately 18 million new cancer cases and 9 million cancer deaths were reported in 2018 (1). Although a substantial amount of work has been undertaken on cancer research and development of numerous drugs for cancer treatment, further investigation is urgently required to identify more specific therapeutic targets as well as drugs with reduced side effects. Currently, extracting new antitumor compounds from traditional medicinal plants is recognized as one of the main cancer treatment strategies (18). However, to date, no study has elucidated whether roburic acid isolated from oak galls exhibits antitumor activity, nor have its interaction target and the underlying mechanisms been identified.

In the present study, we investigated the cytotoxicity of roburic acid in 24 cancer cell lines comprising eight cancer types and identified the colorectal cancer cell lines HCT-116 and HCT-15 as being the most sensitive to roburic acid treatment, with IC_50_ values of 3.90 and 4.77 μM, respectively. These cell lines were then used to further investigate the anticancer effects of roburic acid *in vitro* and *in vivo*. Our results demonstrated that roburic acid effectively suppressed colorectal cancer cell proliferation by inducing G0/G1 cell cycle arrest and downregulating the protein expression of Cyclin B1, Cyclin D1, and Cyclin E1. In addition, roburic acid induced the apoptosis of colorectal cancer cells by promoting the cleavage of PARP, Caspase3, Caspase7, and Caspase9, as well as downregulating the levels of the antiapoptotic proteins Bcl-2, Bcl-xL, XIAP, Mcl-1, and Survivin. Importantly, roburic acid inhibited the TNF-induced NF-κB signaling pathway and suppressed the expression of these antiapoptotic proteins in colorectal cancer cells. Molecular interaction studies further demonstrated that roburic acid directly bound to TNF with high affinity (*K*_D_ = 7.066 μM) and blocked the interaction between TNF and its receptor, TNF-R1. Consistent with the *in vitro* results, roburic acid also suppressed tumor growth by blocking NF-κB signaling in a xenograft mouse model of colorectal cancer.

Recent studies have shown that most natural compounds with anti-inflammatory properties exhibit excellent antitumor activity by inhibiting NF-κB pathway activation (15, 34, 35). The NF-κB signaling pathway is aberrantly activated in many tumor cells, contributing to cancer cell survival, proliferation, differentiation, apoptosis, inflammation, and cell signaling transduction (11, 36, 37). Interestingly, roburic acid was detected in the resin fraction that is secreted when plants are attacked by insects, and it was shown to exhibit anti-inflammatory properties (21, 23), suggesting that it can serve as an insect repellant and might exert protective effects on human health (38). In this study, roburic acid exhibited marked cytotoxic effects in different types of cancer cells (IC_50_ <15 μM), including colorectal, breast, central nervous system, kidney, lung, ovarian, prostate, and leukemia cancer cells. Based on these findings, we speculated that roburic acid likely suppresses cancer cell growth by inhibiting the activation of the NF-κB pathway.

It is well established that a dynamic and complex network of interacting proteins regulates cellular behavior (13). Interactions between TNF with TNF-R1 activate the NF-κB signaling pathway, which plays important roles in cancer development and progression (39). Consequently, targeting protein–protein interactions is a promising strategy for the treatment of cancer (40, 41). TNF is an inflammatory cytokine that initiates dynamic intracellular signals through binding to its receptor TNF-R1 (13). Upon TNF binding, TNF-R1 forms a trimer, which then becomes a key regulator of inflammation-dependent NF-κB signaling. The NF-κB inhibitor protein (IκB) is degraded soon after phosphorylation by activated IκB kinase (IKK), and the p65 transcription factor translocates into the nucleus to activate TNF-induced NF-κB signaling (12, 13). In this study, we found that roburic acid treatment significantly inhibited the TNF-induced phosphorylation of IKKα/β, IκBα, and p65, degradation of IκBα, and nuclear translocation of p65 in human colorectal cancer cells. Regarding the detailed molecular mechanisms, molecular interaction studies demonstrated that roburic acid can directly bind to TNF with high affinity (*K*_D_ = 7.066 μM), but not to its receptor, TNF-R1. Interestingly, roburic acid also blocked the interaction between TNF and TNF-R1, with a decrease in binding affinity (*K*_D_) from 7.33 to 211 nM, which explains why roburic acid can inhibit the TNF-induced NF-κB signaling pathway. However, the mechanisms underlying how roburic acid binds to TNF remain unclear and require further investigation.

Apoptosis is the process of programmed cell death, and plays a pivotal role in the development and progression of cancer (16). Excessive NF-κB signaling in cancer cells can suppress apoptosis *via* inducing the expression of apoptosis inhibitors such as XIAP, Mcl-1, and Survivin (10, 12). In this study, we demonstrated that roburic acid suppressed the TNF-induced expression of antiapoptotic proteins, including XIAP, Mcl-1, and Survivin. Without exogenous TNF stimulation, roburic acid also inhibited the expression of these antiapoptotic proteins in colorectal cancer cells. NF-κB can promote cell proliferation by transactivating the expression of Cyclin D1 and c-Myc (10). In the current study, we found that roburic acid could significantly downregulate Cyclin D1 and c-Myc protein levels in colorectal cancer cells, with or without TNF stimulation. *In vitro* studies have established that roburic acid can effectively suppress human colorectal cancer cell growth through inhibition of the NF-κB signaling pathway; however, whether roburic acid also exhibited antitumor activity *in vivo* through the same mechanism has not been clarified. In the present study, we further demonstrated that roburic acid can also inhibit tumor growth *in vivo* by blocking NF-κB signaling in a xenograft mouse model of colorectal cancer.

Roburic acid is a natural small-molecule compound isolated from medicinal plants, and its biological activities and mechanism of action have not been thoroughly studied. Additionally, roburic acid may have many limitations, such as poor water solubility and bioavailability, and potential side effects. Therefore, these issues need to be urgently solved before roburic acid can be used clinically in the treatment of patients with colorectal cancer. Notably, in addition to contributing to cancer development and progression, TNF/TNF-R1-mediated NF-κB signaling has crucial roles in many other autoimmune diseases such as rheumatoid arthritis and Crohn’s disease (17, 42, 43). Based on the findings in this study, we speculate that roburic acid could also be used in the treatment of other TNF-related diseases. However, further investigation is urgently needed to test this hypothesis, which is the subject of ongoing work in our laboratory.

## Conclusions

Taken together, our findings showed that roburic acid directly binds to TNF with high affinity, thereby disrupting the interaction between TNF and TNF-R1 and leading to inhibition of the NF-κB signaling pathway, both *in vitro* and *in vivo*. The results indicated that roburic acid is a novel TNF-targeting therapeutics agent in colorectal cancer as well as other cancer types.

## Data Availability Statement

The original contributions presented in the study are included in the article/**Supplementary Material**. Further inquiries can be directed to the corresponding authors.

## Ethics Statement

This study was carried out in accordance with the National Institutes of Health’s Guidelines for the Care and Use of Laboratory Animals and was approved by the Yunnan Agricultural University institutional ethics committee.

## Author Contributions

HX, TL, and ZX conceived and designed the experiments. TL, HX, JL, FC, JX, LH, and LJ performed the experiments. HX and TL analyzed the data. JS and XW contributed reagents/materials/analysis tools. HX and TL wrote the manuscript. All authors read and approved the final manuscript.

## Funding

This work was supported by grants from the Scientific Research Fund Project of Yunnan Provincial Education Office (2022J0297), the Yunnan Fundamental Research Project (202101AU070216, 202101AU070086, and 202101AT070749), the Yunnan Provincial Key Programs of Yunnan Eco-friendly Food International Cooperation Research Center Project (2019ZG00904 and 2019ZG00909), and the Science and Technology Plan Project of Yunnan Province (2018IA060).

## Conflict of Interest

The authors declare that the research was conducted in the absence of any commercial or financial relationships that could be construed as a potential conflict of interest.

## Publisher’s Note

All claims expressed in this article are solely those of the authors and do not necessarily represent those of their affiliated organizations, or those of the publisher, the editors and the reviewers. Any product that may be evaluated in this article, or claim that may be made by its manufacturer, is not guaranteed or endorsed by the publisher.

## Abbreviations

BLI, biolayer interferometry; BSA, bovine serum albumin; DMSO, dimethyl sulfoxide; FBS, fetal bovine serum; IC_50_, half-maximal inhibitory concentration; *K*_D_, binding affinity; *K*_off_, dissociation constant; *K*_on_, association constant; MTT, 3-(4,5-dimethylthiazol-2-yl)-2,5-diphenyltetrazolium bromide; NF-κB, nuclear factor-kappa B; PARP, poly (ADP-ribose) polymerase; PBS, phosphate-buffered saline; PI, propidium iodide; PMSF, phenylmethylsulfonyl fluoride; P/S, penicillin–streptomycin solution; PVDF, polyvinylidene fluoride; RIPA, radioactive immunoprecipitation assay; SEM, standard error of the mean; SPR, surface plasmon resonance; SSA, super streptavidin; TNF, tumor necrosis factor; TNF-R1, TNF receptor 1.
