# Supplementary material for: Roburic Acid Targets TNF to Inhibit the NF-κB Signaling Pathway and Suppress Human Colorectal Cancer Cell Growth
Source: Front Immunol. 2022 Feb 9;13:853165. doi: 10.3389/fimmu.2022.853165 (PMC8864141; doi:10.3389/fimmu.2022.853165)
Supplement: Supplementary file 1 [file DataSheet_1.pdf]

Supplementary Materials

Roburic Acid Targets TNF to Inhibit the NF-κB Signaling Pathway and Suppress Human Colorectal Cancer Cell Growth

Huanhuan Xu[1,2,†], Titi Liu[1,2,†], Jin Li[1,2], Fei Chen[1,2], Jing Xu[1], Lihong Hu[1], Li Jiang[1], Zemin Xiang[1,2,*], Xuanjun Wang[1,2,3,*] and Jun Sheng[1,3,*]

1. Key Laboratory of Pu-er Tea Science, Ministry of Education, Yunnan Agricultural University, Kunming 650201, China

2. College of Science, Yunnan Agricultural University, Kunming 650201, China

3. State Key Laboratory for Conservation and Utilization of Bio-Resources in Yunnan, Kunming 650201, China

[†]These authors contributed equally to this work.

[*]Correspondence to: Key Laboratory of Pu-er Tea Science, Ministry of Education, Yunnan Agricultural University, No. 452, Fengyuan Road, Panlong District, Kunming 650201, China.

E-mail addresses: xiangzmwdx@sohu.com (Zemin Xiang), jwang@ynau.edu.cn (Xuanjun Wang), shengj@ynau.edu.cn (Jun Sheng).

Table S1. The IC_{50} values (μM) of roburic acid in 16 cancer cell lines

Cancer types	Cell lines	IC_{50} values (μM)
Breast	SK-BR-3	7.46
	BT549	10.99
	BT-474	14.57
CNS	U251	6.75
Kidney	786-O	5.78
	ACHN	6.14
	A498	12.37
Lung	A549	5.91
	NCI-H460	7.65
	NCI-H226	14.42
	NCI-H23	8.55
Ovary	OVCAR-3	6.63
	SK-OV-3	5.43
Prostate	DU145	7.89
	PC-3	9.67
Leukemia	CCRF-CEM	5.45

The cancer cells were incubated with increasing concentrations of roburic acid for 48 h. Cell viability was determined by MTT assay and IC_{50} values were calculated.

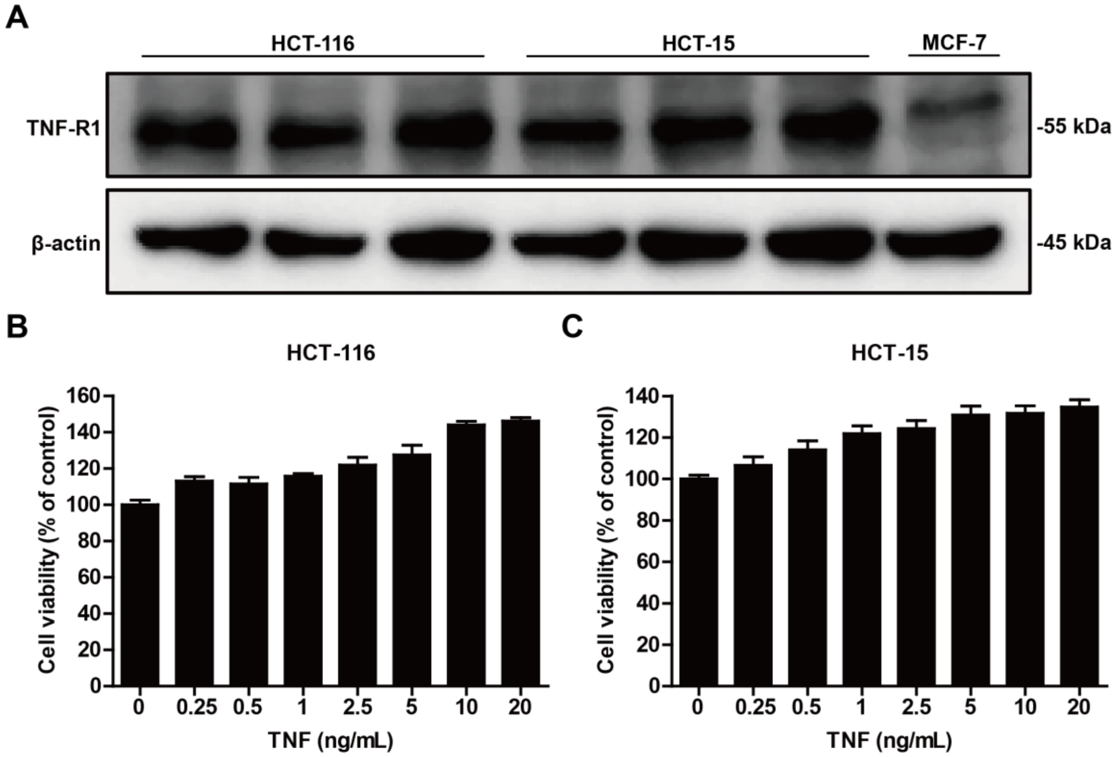

Figure S1. TNF promotes the viability of human colorectal cancer cells. (**A**) The expression levels of TNF-R1 were detected in the HCT-116, HCT-15, and MCF-7 cell lines. HCT-116 (**B**) and HCT-15 (**C**) cells were treated with various concentrations of TNF (0.25–20 ng/mL) for 48 h. An MTT assay was performed to evaluate cell viability. Representative images are displayed. Data are shown as means ± SEM of three independent replicates.

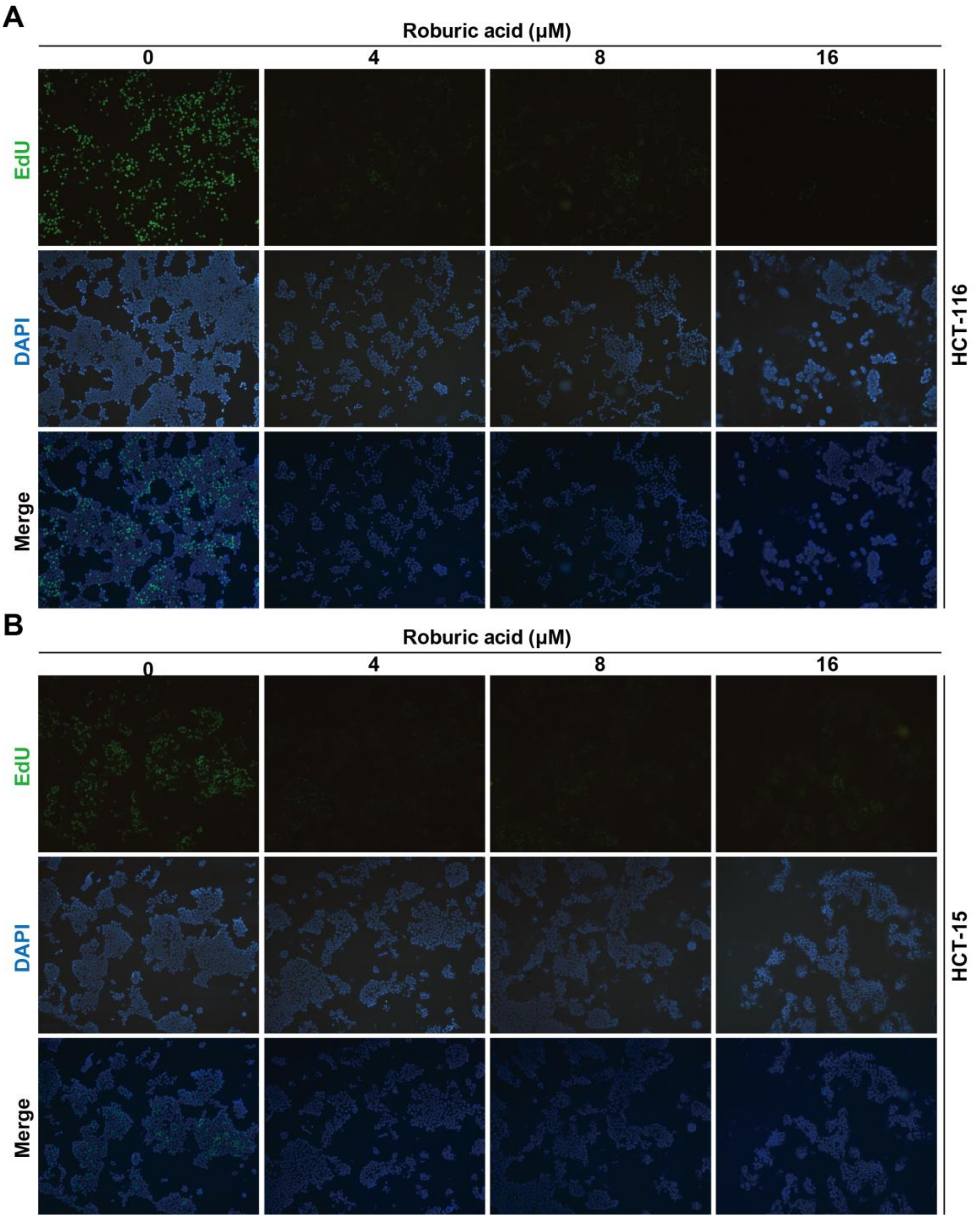

Figure S2. Roburic acid inhibits DNA synthesis in HCT-116 and HCT-15 cells. HCT-116 (**A**) and HCT-15 (**B**) cells were treated with various concentrations of roburic acid (4, 8, or 16 μM) for 24 h. DNA synthesis was determined by EdU incorporation assay. Images (original magnification ×200) are representative of three independent experiments.

A

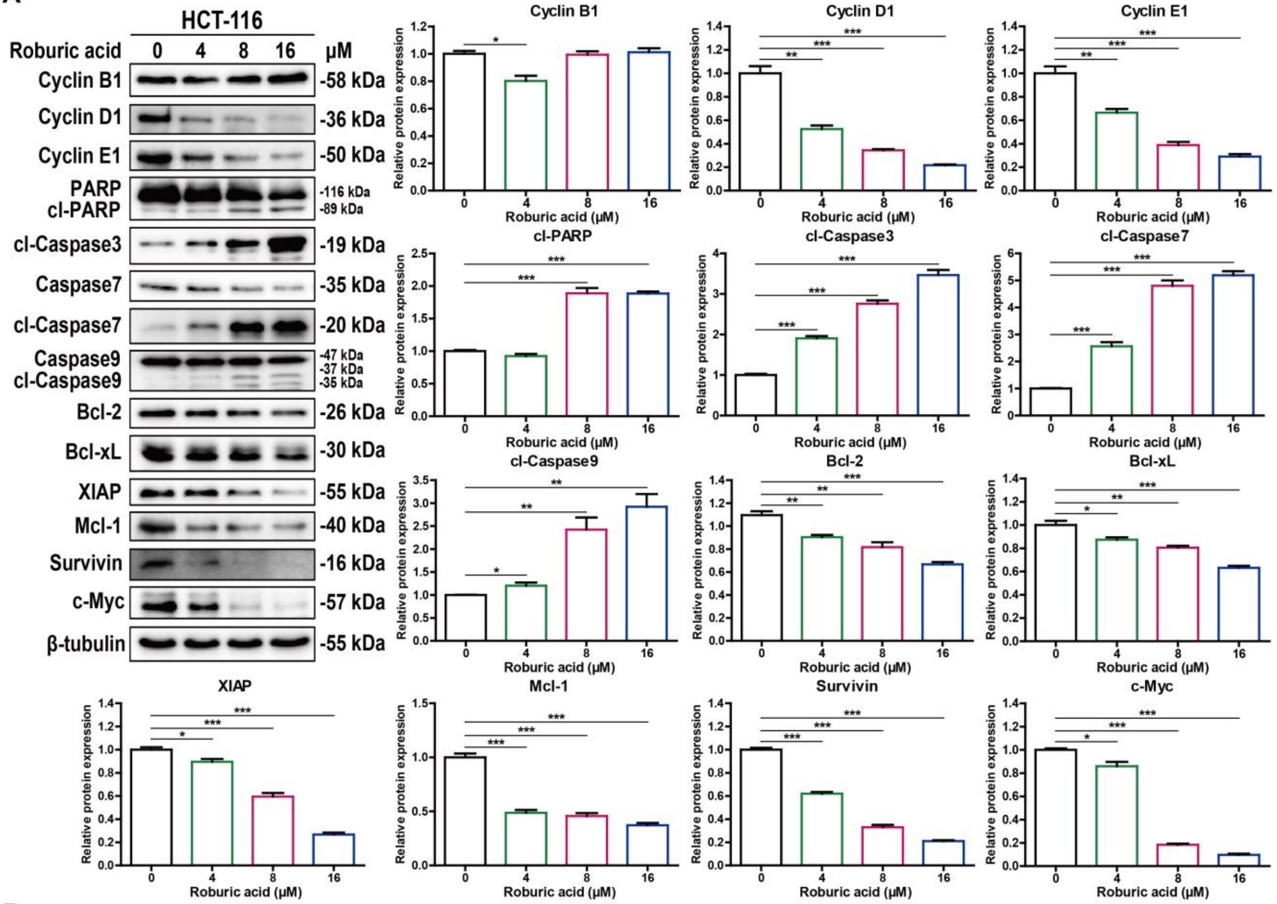
HCT-116
Roburic acid 0 4 8 16 μM
Cyclin B1 -58 kDa
Cyclin D1 -36 kDa
Cyclin E1 -50 kDa
PARP -116 kDa
cl-PARP -89 kDa
cl-Caspase3 -19 kDa
Caspase7 -35 kDa
cl-Caspase7 -20 kDa
Caspase9 -47 kDa
cl-Caspase9 -37 kDa -35 kDa
Bcl-2 -26 kDa
Bcl-xL -30 kDa
XIAP -55 kDa
Mcl-1 -40 kDa
Survivin -16 kDa
c-Myc -57 kDa
β-tubulin -55 kDa
Cyclin B1
Cyclin D1
Cyclin E1
cl-PARP
cl-Caspase3
cl-Caspase7
cl-Caspase9
Bcl-2
Bcl-xL
XIAP
Mcl-1
Survivin
c-Myc
Relative protein expression
Roburic acid (μM)

B

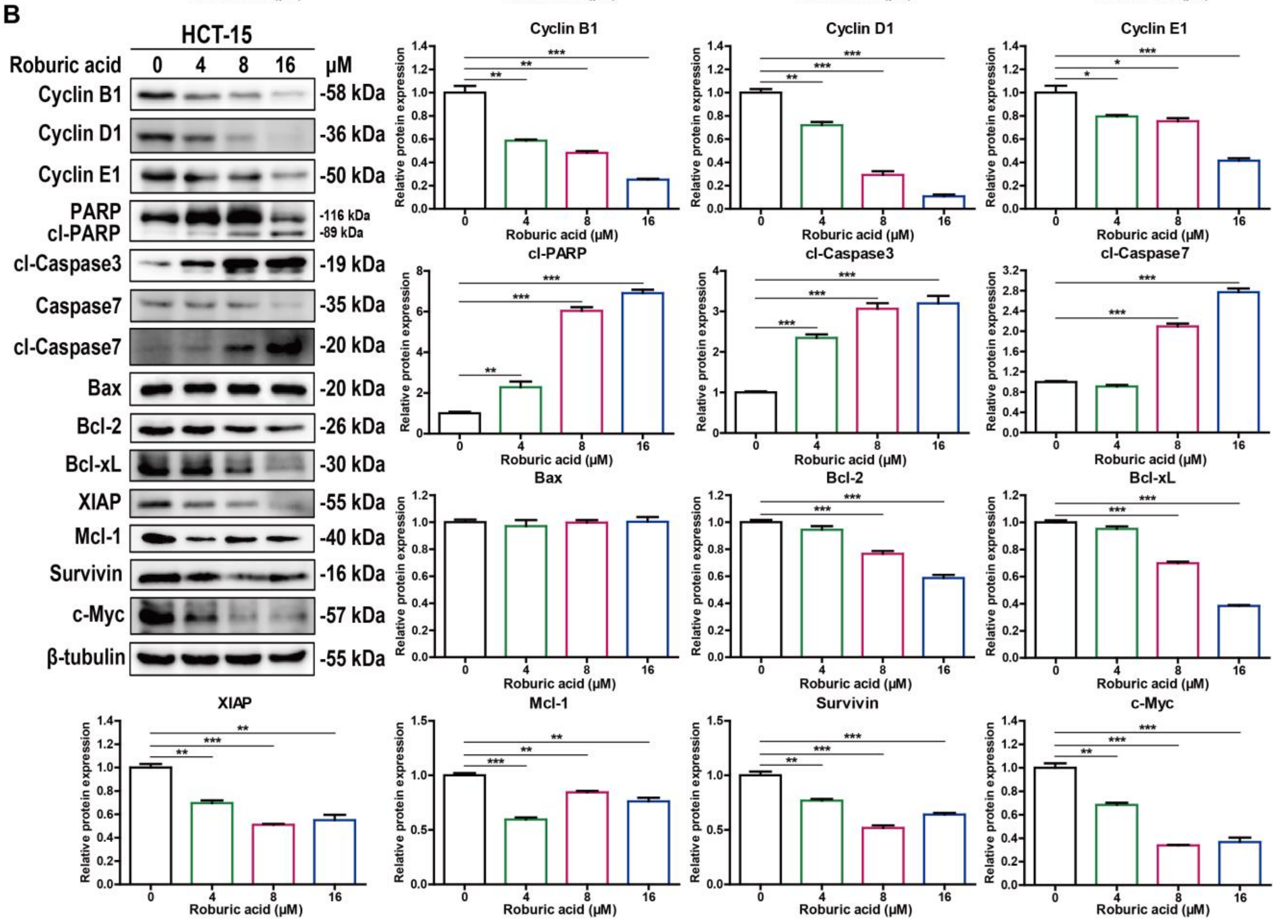
HCT-15
Roburic acid 0 4 8 16 μM
Cyclin B1 -58 kDa
Cyclin D1 -36 kDa
Cyclin E1 -50 kDa
PARP -116 kDa
cl-PARP -89 kDa
cl-Caspase3 -19 kDa
Caspase7 -35 kDa
cl-Caspase7 -20 kDa
Bax -20 kDa
Bcl-2 -26 kDa
Bcl-xL -30 kDa
XIAP -55 kDa
Mcl-1 -40 kDa
Survivin -16 kDa
c-Myc -57 kDa
β-tubulin -55 kDa
Cyclin B1
Cyclin D1
Cyclin E1
cl-PARP
cl-Caspase3
cl-Caspase7
Bax
Bcl-2
Bcl-xL
XIAP
Mcl-1
Survivin
c-Myc
Relative protein expression
Roburic acid (μM)

Figure S3. Roburic acid modulates the expression levels of multiple cell cycle- and apoptosis-related regulators in colorectal cancer cells. HCT-116 (**A**) and HCT-15 (**B**) cells were treated with roburic acid (4, 8, or 16 μM) for 24 h. Then, the collected cell lysates were used for immunoblotting analysis to measure the expression of the indicated proteins. Beta-tubulin was used as the loading control and the gray densities of bands corresponding to the indicated proteins were quantified using AlphaView software. Representative images are displayed. Data are shown as means ± SEM of three independent replicates. $^{*}P < 0.05$, $^{**}P < 0.01$, and $^{***}P < 0.001$ compared with the control.

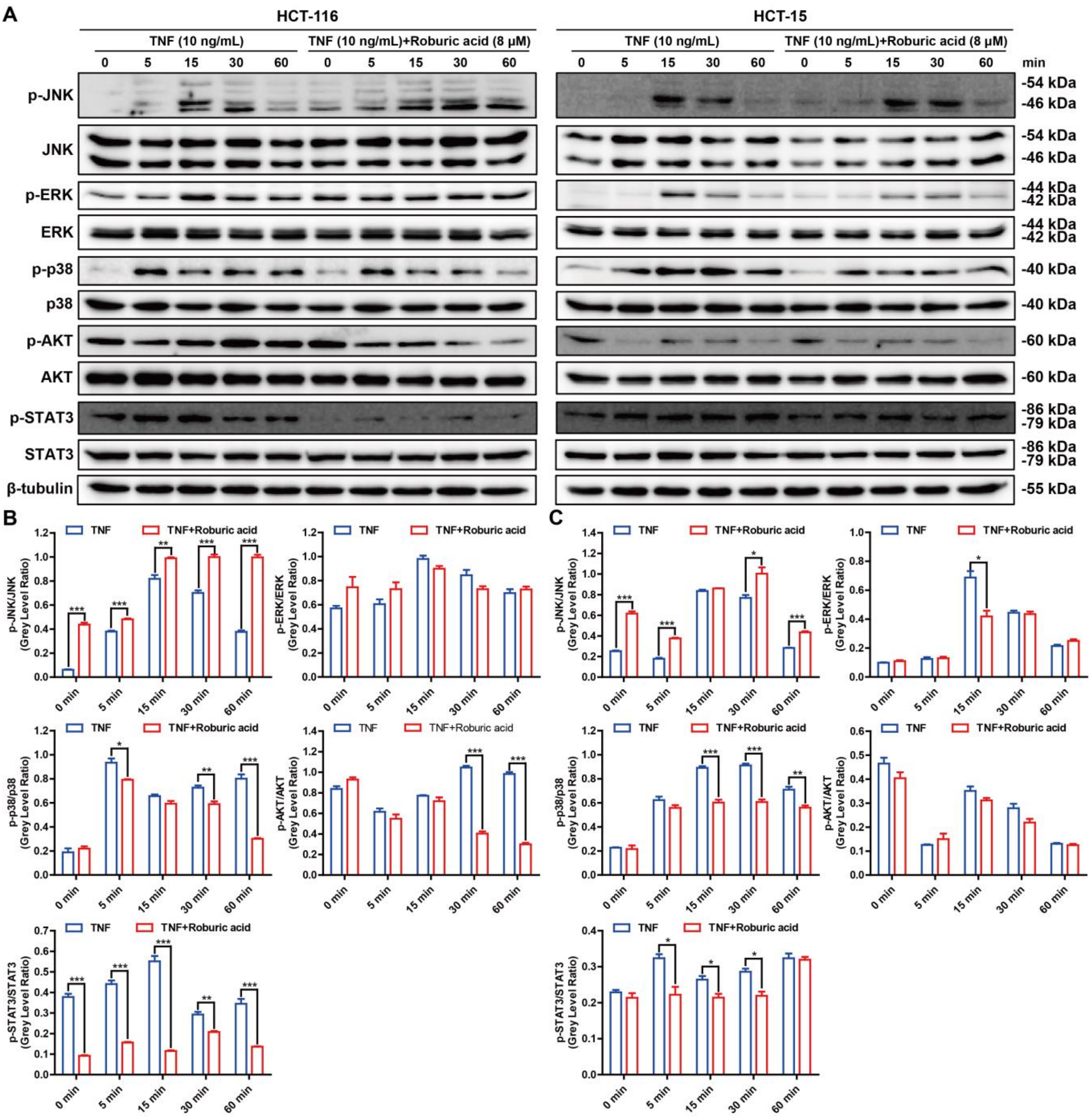

Figure S4. Roburic acid modulates the TNF-induced phosphorylation of MAPK, AKT, and STAT3 in colorectal cancer cells. (**A**) HCT-116 and HCT-15 cells were treated with roburic acid and TNF as described in **Figure 5**. Cell lysates were subjected to western blotting using the indicated primary antibodies. The gray densities of the bands corresponding to the indicated proteins in HCT-116 (**B**) and HCT-15 (**C**) cells were quantified using AlphaView software. Representative images

are displayed. Data are shown as means ± SEM of three independent replicates. Asterisks indicate significant differences compared with TNF treatment at the same time point ($^{*}P < 0.05$, $^{**}P < 0.01$, and $^{***}P < 0.001$).

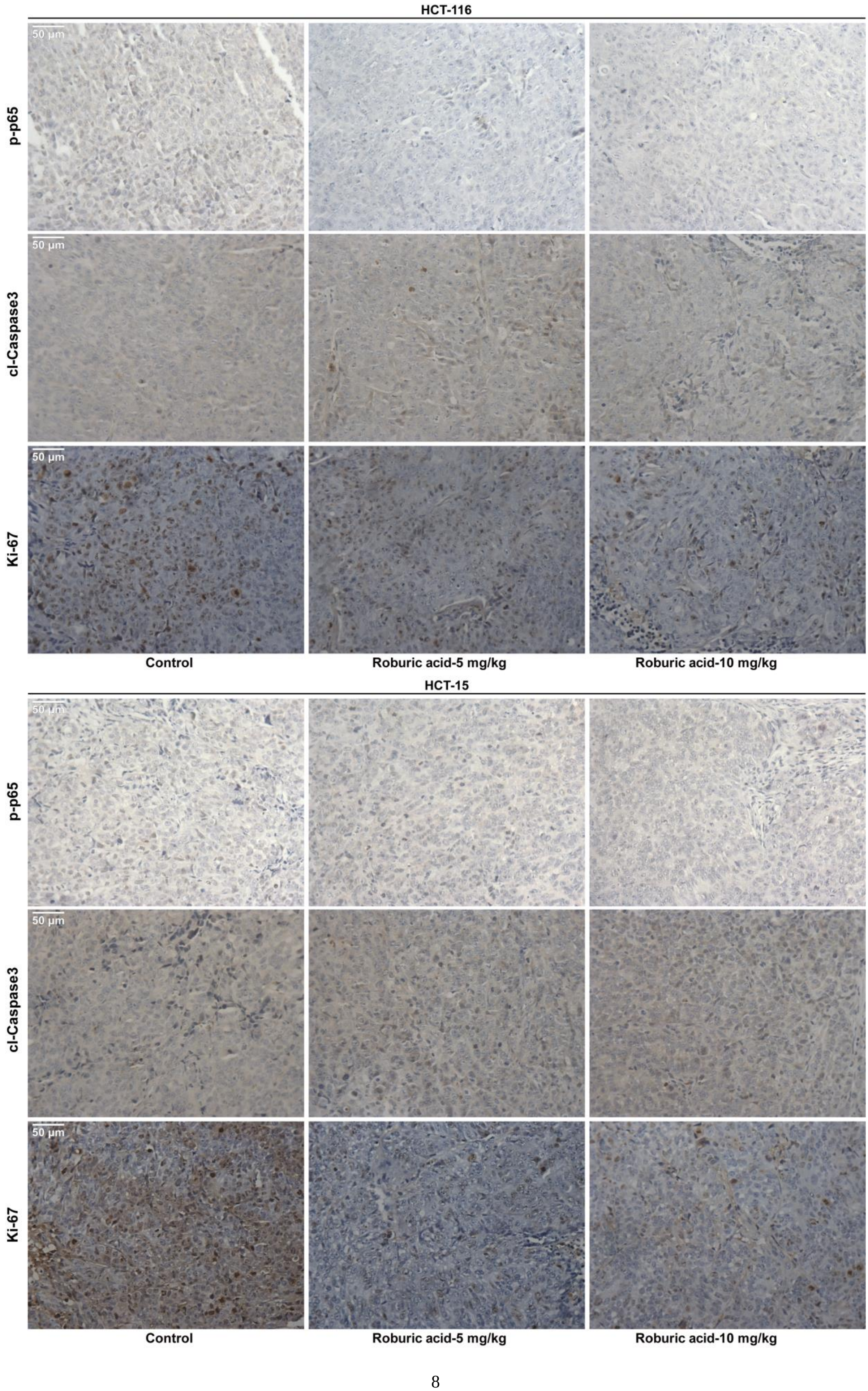
HCT-116
50 μm
p-p65
cl-Caspase3
Ki-67
Control
Roburic acid-5 mg/kg
Roburic acid-10 mg/kg
HCT-15
p-p65
cl-Caspase3
Ki-67
Control
Roburic acid-5 mg/kg
Roburic acid-10 mg/kg

Figure S5. Paraffin-embedded HCT-116 and HCT-15 tumor tissue sections were immunostained with antibodies against p-p65, cl-Caspase3, and Ki-67 (original magnification ×400).
